# Chicago Medical Society

**Published:** 1878-04

**Authors:** 


### CHICAGO MEDICAL SOCIETY.

Regular meeting, Monday, March 4, 1878. President Dr. E. Ingals in the chair; 20 members present.

*Methods of measuring the lower extremities.*—Dr. John Bartlett read a paper in which he pointed out the defects of the commonly adopted modes of measuring the length of the legs. He then exhibited a new apparatus which makes the measurements perfectly reliable; he explained the principles upon which the apparatus was constructed, described its construction, and demonstrated its application.*

Regular meeting, Monday, March 18, 1878. President Dr. E. Ingals in the chair; 32 members present.

Dr. T. D. Fitch read the *Report on Obstetrics*, dwelling at some length upon the following subjects:

1. *Use of Ancæsthetics in Labor.*—This question was thoroughly ventilated at the meeting of the American Gynæcological Society last year, and at the meeting of the Detroit Medical Journal Association. The discussion on both occasions revealed the fact that the leading obstetricians are far from being unanimous in their opinion regarding the use of chloroform or ether—the harmlessness or injuriousness of anæsthesia. The essayist's view of the question is that anæsthesia, partial or complete, has a strong tendency to diminish the force and frequency of uterine contractions, or of abolishing them entirely; that it also abolishes the contractions of the abdominal muscles and diaphragm which materially aid in the expulsion of the fœtus. Anæsthetics materially interfere with the third stage of labor, and predispose to post-partum hemorrhage. "I have had sufficient experience in this matter to convince me that they should not be used under any circumstances in this stage of labor, not even when manual delivery of the placenta has to be

---

* We shall publish the full description, with illustrations, in an original communication on this subject by Dr. Bartlett in our next issue.—Ed.

resorted to." He prefers ether to chloroform, and gives an alcoholic stimulant from fifteen to thirty minutes before the anæsthetic.

2. *Hot water as a hæmostatic.*—Considerable has lately been written on injections of hot water to arrest uterine hemorrhages. Instances from American, French and German literature were quoted to show the successful application of hot water in metrorhagia. The writer's experience fully confirmed these reports. He tried it in two cases of abortion, the flowing was excessive and the patients were almost pulseless. The hot water acted like magic; in a few moments all flowing ceased and in less than half an hour the fœtus was expelled. Both patients made excellent recoveries. In eight cases of menorrhagia in which the flowing was very profuse, the hot water injections proved most beneficial, in every case controlling the flowing. These hot water injections (105 degrees F.) are more pleasant to the patient than the cold water, and never cause any dangerous reaction.

3. *Management of cases of abortion, especially of its third stage.*—On this subject a very interesting debate took place at a meeting of the New York obstetrical society in last February. The management favored by the majority was to dilate the os uteri, and then give ergot to expel the fœtus. As to the placenta if it could be easily removed, let it be removed at once; if adherent and not removable without too much force, let the tampon be used and ergot given for several days; if that was not sufficient, then dilate the os and remove placenta, using disinfectants afterwards. Dr. Fitch always first makes an examination and inspects the discharges to know whether the ovum or placenta, or both, have been discharged. If flowing is slight and os undilated, enjoin rest, empty the bowels and give an opiate. If flowing is profuse, and os undilated, use tampon and ergot; if os is dilatable, dilate with finger and remove ovum with finger or hook or forceps if you can feel it; but if you cannot reach any part of the ovum with your finger, do not work in the dark, fishing for it with instruments, but give ergot and let the uterus alone.

The discussion which followed revealed the fact that in regard to the use of anæsthesia in labor cases and in regard to the management of abortion, there exists as much diversity of opinion among western practitioners, as among their eastern brethren. But the hæmostatic effect of hot water injections was confirmed by every one who had tried them.

The committee appointed at a previous meeting to confer with the Society of Physicians and Surgeons regarding a union of the two societies, reported that a conference was held which resulted in making to both societies the following propositions: The Chicago Medical Society shall hold its meetings at a central locality; the members of the society of Physicians and Surgeons not already members of the Chicago Medical Society shall become members of the latter society without initiation fee; reorganization of the working plan of the society.

A very animated debate took place; but final action on these propositions was deferred.

---

THERE are two different states found in women where iron is either totally contra-indicated or to be given with great caution. The first is a condition of amenorrhœa in florid, plethoric persons. The other is the opposite condition of menorrhagia in certain females. There are cases of menorrhagia associated with pallor and debility, where the usual compound of iron and extract of ergot is not so useful as a non-chalybeate treatment. In these cases it is not any imperfection in the process of blood manufacture which is to be remedied, for the blood is made rapidly and quickly, only to be lost at each menstrual period. It is here desirable rather to limit the rapidity of the blood formation, so that when the several vascular turgescence of the menstrual period comes, it will not find the blood vessels too distended with blood. This will lead to diminished catamenial loss, and so the blood waste will be economised. According to the experience of Dr. Brown Séquard and Dr. Hughlings Jackson, iron does not suit epileptics. It increases the tendency to fits. It may improve the general condition, but it aggravates the epilepsy.—*Dublin Medical Press*, Oct. 3.

